# Wikipedia Chemical Structure Explorer: substructure and similarity searching of molecules from Wikipedia

**DOI:** 10.1186/s13321-015-0061-y

**Published:** 2015-03-22

**Authors:** Peter Ertl, Luc Patiny, Thomas Sander, Christian Rufener, Michaël Zasso

**Affiliations:** Novartis Institutes for BioMedical Research, Novartis Campus, CH-4056 Basel, Switzerland; Ecole Polytechnique Fédérale de Lausanne (EPFL), Institute of Chemical Sciences and Engineering (ISIC), 1015 Lausanne, Switzerland; Actelion Pharmaceuticals Ltd., Gewerbestrasse 16, CH-4123 Allschwil, Switzerland

**Keywords:** Wikipedia, SMILES, Substructure search, Similarity search, Chemical database, JavaScript

## Abstract

**Background:**

Wikipedia, the world’s largest and most popular encyclopedia is an indispensable source of chemistry information. It contains among others also entries for over 15,000 chemicals including metabolites, drugs, agrochemicals and industrial chemicals. To provide an easy access to this wealth of information we decided to develop a substructure and similarity search tool for chemical structures referenced in Wikipedia.

**Results:**

We extracted chemical structures from entries in Wikipedia and implemented a web system allowing structure and similarity searching on these data. The whole search as well as visualization system is written in JavaScript and therefore can run locally within a web page and does not require a central server. The Wikipedia Chemical Structure Explorer is accessible on-line at www.cheminfo.org/wikipedia and is available also as an open source project from GitHub for local installation.

**Conclusions:**

The web-based Wikipedia Chemical Structure Explorer provides a useful resource for research as well as for chemical education enabling both researchers and students easy and user friendly chemistry searching and identification of relevant information in Wikipedia. The tool can also help to improve quality of chemical entries in Wikipedia by providing potential contributors regularly updated list of entries with problematic structures. And last but not least this search system is a nice example of how the modern web technology can be applied in the field of cheminformatics.

**Graphical abstract:**
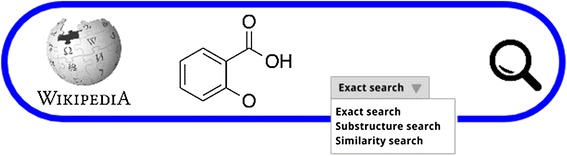
Wikipedia Chemical Structure Explorer allows substructure and similarity searches on molecules referenced in Wikipedia.

## Background

Wikipedia is the 6^th^ most accessed web site worldwide [1] with currently more than 4.6 million articles in English and many million in other languages. It is an indispensable reference for all scientific disciplines, among others also for chemistry [2], containing information about numerous important molecules including metabolites, drugs, agrochemicals, industrial chemicals and many others. Wikipedia contains currently entries for over 15,000 chemicals, growing by about 1000 new molecules per year. These chemical pages have been created and are constantly updated by thousands of enthusiastic Wikipedia users, both experts in the field and random passersby. Without this huge volunteer effort the tool we are presenting here would not be possible. The authors want therefore to express their gratitude to the Wikipedia community and contribute to this global effort by developing and making freely available the Wikipedia Chemical Structure Explorer. Our motivation was to address lack of support for special chemical searches in Wikipedia. The tool we are presenting here offers an easy way to perform substructure and similarity searches for molecules referenced in Wikipedia.

## Implementation

### Extraction of molecules from Wikipedia

**Figure 1:**
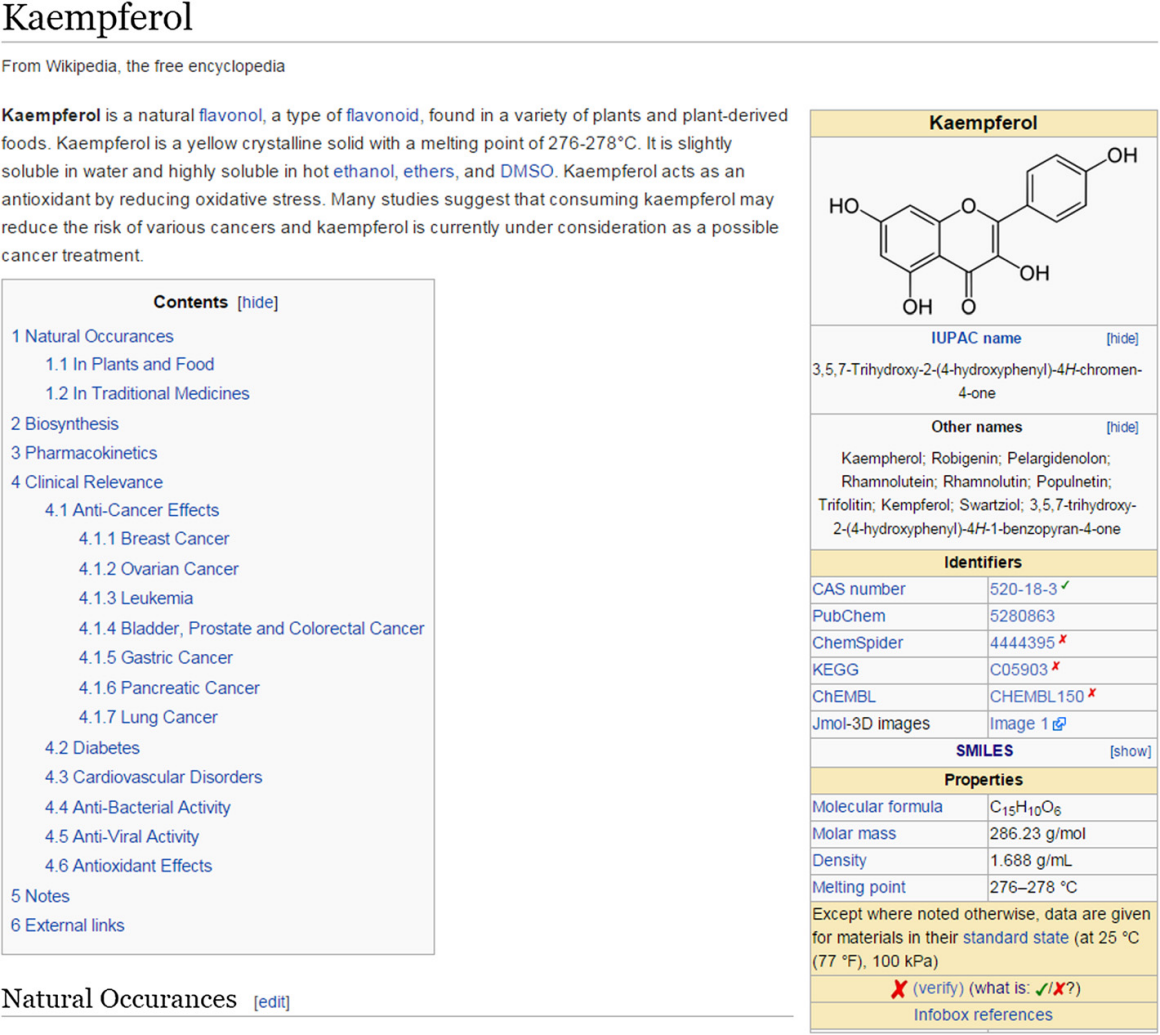
**A Wikipedia page with example of a Chembox template.**

**Figure 2:**
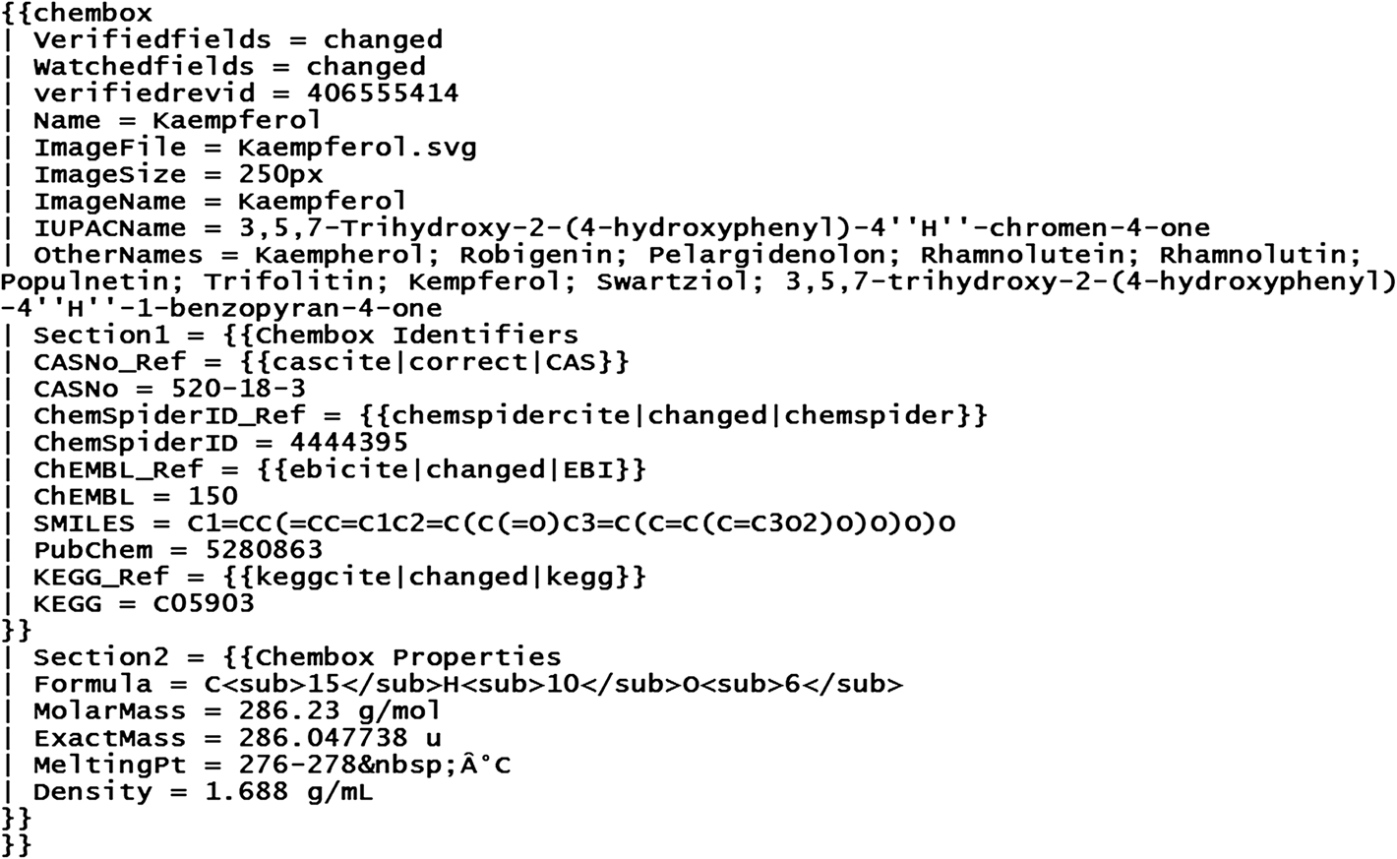
**An example of a Chembox in Wiki markup format.**

Among other chemical content Wikipedia contains numerous entries describing specific chemicals. Such entries are using a special chemical template [3], either an “Infobox drug” (also called Drugbox) used for drugs or a Chembox used for other chemicals. These chemical templates (also called infoboxes) are pieces of Wikipedia markup embedded into chemistry pages that contain the most important information about molecules, allowing to present chemical data in a standardized way and support also computer mining of the data. The templates have a modular design. After general information including chemical name and structure depiction, possibly also a 3D molecule image, SMILES code [4] and links to other chemical databases like PubChem [5] or ChEMBL [6] these boxes often contain also other data like physicochemical and pharmacological properties, information about chemical hazard and so on. They can be built from multiple sections, each covering a group of information. Depending on the compound, sections can be added or left out, and within a section parameters can be added or omitted. An example of Wikipedia page with a Chembox is shown in Figure 1 and its encoding in Wiki markup in Figure 2.

Several projects under Wikipedia umbrella are using information stored in the chemical infoboxes. One of them is DBpedia:Chemical Compound project [7] extracting structured information from Wikipedia, converting it into RDF format and making it freely available on the Web. DBpedia is therefore something like the Semantic Web mirror of Wikipedia. Another activity in this area is the Wikidata:WikiProject Chemistry [8]. Wikidata is a free database of the structured data extracted from Wikipedia. Its Chemistry subproject focuses on defining data items for chemical entries and checking their quality and also organizes and monitors the data implementation (also with automatic bots).

Wikipedia maintains list of all entries containing either Chembox or Drugbox template [9] what makes identification of the pages describing molecules relatively easy. These lists can be accessed through the MediaWiki API in a JSON format. To extract chemical structures from infoboxes we developed a JavaScript program executed on the Node.js [10] platform to process those lists and then download the pages in their “raw” (Wiki markup) format. The content of chemical infoboxes from 15,474 downloaded pages was then extracted and parsed to obtain the SMILES codes. We found that many molecular entries (about 2200) do not contain any SMILES at all, for example pages for mixtures, complex substances (like Nitrocellulose), antibodies, polymers and so on. Also many entries of “normal” molecules do not contain SMILES because it was simply not entered by the authors creating and editing the article. In cases that a page contained more than one SMILES, they were all parsed. If these SMILES codes represent exactly the same structure, only the first one is kept. When the structures are different (usually due to inclusion of stereochemical information), an entry is created for each SMILES.

At the end we were able to extract some 13,000 SMILES codes for the Wikipedia entries. Over 600 of these codes could not be processed by the SMILES parser. A clear majority of the problems (over 350 cases) was caused by not respecting the SMILES syntax rules for unsubstituted pyrrole-type nitrogen. This nitrogen was encoded as n and not as [nH] as required by the SMILES grammar (so for example benzimidazole was incorrectly encoded as n2c1ccccc1nc2). Since the incorrect SMILES encoding of many heteroaromatic molecules is apparently an issue in Wikipedia we recommend entering SMILES to Chemboxes or Drugboxes in its nonaromatic form with alternating single and double bonds. Other SMILES errors were caused by missing ring closures, unclosed parentheses, hydrogens and non-organic atoms outside square brackets and so on.

In the process of extraction the authors (LP and MZ) corrected over 100 such errors directly in Wikipedia. Still remaining SMILES codes that cannot be parsed may be viewed after clicking the “Browse errors” link on the Wikipedia Explorer main page. This list of problematic SMILES’s is generated regularly on a daily basis. A click on the SMILES code opens directly the respective Wikipedia page. We hope that this mechanism will make correction of SMILES errors for the Wikipedia cheminformatics community much easier.

As mentioned previously, the chemical infoboxes contain in addition to SMILES also links to other chemical databases, most notably to PubChem [5]. When the Wikipedia entry contained both the SMILES code and the link to PubChem it was possible to check whether both structures are the same. In over 600 cases we could see that these structures differ. In most cases this was caused by difference in tautomeric form but in many cases the two molecules differed also in position of functional groups, or atom types, caused probably by errors when drawing the structures. In some cases the two structures differ completely (probably inclusion of an incorrect PubChem CompoundID). In such ambiguous cases we used structures from Wikipedia but disagreement with the PubChem may be indication of problems that should be checked in the future.

**Figure 3:**
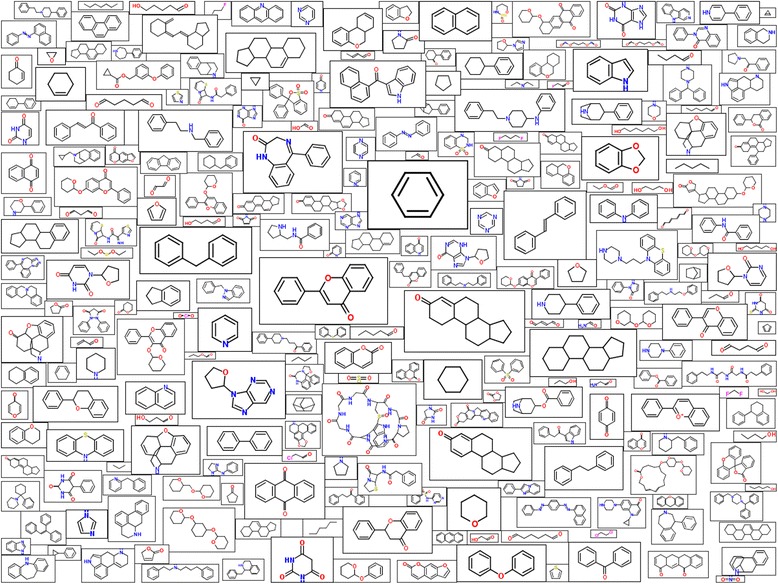
**The 250 most common scaffolds of molecules from Wikipedia presented as a Molecule Cloud diagram [** 11 **] documenting diversity of entries.**

After final processing the database of SMILES codes of Wikipedia molecules contained 13,072 entries. To document diversity of this molecule collection the 250 most frequent scaffolds present in this set are shown in Figure 3 in form of a Molecule Cloud diagram [11]. The size of the scaffold image is proportional to the number of molecules containing this scaffold, ranging from the largest benzene (there are 1116 entries for benzene derivatives in Wikipedia) down to the smallest images representing 5 Wikipedia entries. The 250 scaffolds displayed in the Figure 3 represent together 4294 Wikipedia molecules. Although detailed analysis of Wikipedia chemical content is out of scope of this communication, it is interesting to compare at least briefly the Wikipedia scaffolds with those present in the common synthetic molecules and bioactive molecules (Figures four and five in ref. [11]). In Wikipedia one can see clear preference for more complex structures, like structures of natural products, steroids or cores of common drugs. This is, of course, nothing surprising, because the Wikipedia chemical entries are created subjectively based on the usefulness and application area of the respective molecules.

The list of entries with canonical SMILES also allows an easy check of structure duplication. We could identify about 30 cases where different Wikipedia pages describe the same molecules (molecules with the same SMILES). This is caused by different spelling (Dichlorophen vs Dichlorophene) but mostly by use of synonyms (for example Amphetamine vs Adderall, Pozanicline vs A-84,543, or Tretinoin vs Retinoic_acid). Some of these mismatches are caused by not including the stereochemical information in SMILES (three sugars with different stereochemistry, namely Pinitol, Quebrachitol and Ononitol are described by the same nonstereo SMILES), and some clearly by error (for example 2-Nitropropane and 1-Nitropropane entries both have the same SMILES).

The data extraction procedure is currently set-up to run automatically every day, so the Wikipedia Explorer contains always the actual information. The “About” page provides information about the latest update and the number of structures extracted. The extracted data (SMILES codes and names of the respective Wikipedia pages) may be obtained by clicking the “Download SMILES” link in the Explorer top menu or downloaded from www.cheminfo.org/wikipedia/smiles.txt.

### Substructure and similarity searching

As mentioned in the introduction, the main motivation that prompted us to embark on the present project was to offer possibility of easy substructure and similarity searches for chemical entries in Wikipedia. Majority of classical chemical search engines currently in use rely on a central server and therefore have numerous susceptibilities, not only possible server malfunction but also in the more extreme scenario of abandonment of the project with resulted end of maintenance and updates. One approach to prevent this risk is to release the server code as an open-source but to install an open source software from scratch usually requires relatively good IT skills. A more recent approach is to distribute directly a server image [12] that allows to set up quickly a new server using a virtual machine. In this project we decided to use this approach and capitalize on the latest web technologies to warrant the project sustainability by moving all the search intelligence from the server to the client. As a result we are able to make exact search, substructure and similarity search using pure HTML5 technology, compatible with most of the recent browsers [13] without any access to an external web service.

The Wikipedia Explorer code was developed based on the open source *Visualizer* project available on the GitHub [14]. The *Visualizer* is a modular web framework written in JavaScript intended for development of scientific applications with particular focus on chemistry. This system allows to display and process various types of information like maps, matrices, chemical structures, spectra or images. It unifies many JavaScript components that are useful for chemists like JSME molecule editor [15], JSmol 3D structure visualizer [16], jsGraph tool for graphics visualization [17], jsNMR for visualization of NMR spectra [18], or various useful machine learning functions [19]. Several applications using the *Visualizer* toolkit can be found on the website www.cheminfo.org. Creating a new application in the *Visualizer* is simple and requires only two files written in the JSON format. One file containing the data and one file describing the modules, their position and their interactions. Therefore any future update of the Wikipedia Chemical Structure Explorer will require only modification of one of these files.

In our system all molecules from Wikipedia are stored in the JSON data file as an array of objects. Each object contains a canonical representation of the chemical structure, an array of 16 numbers containing the 512 index bits used for the structure similarity searching and substructure fingerprint pre-screening, the molecular formula, the molecular weight as well as the Wikipedia page name allowing the client to create a direct link to the original information. This file is currently 5 MiB large, but as httpd servers and web browser negotiate compression algorithm, the transferred data are actually less than 2 MiB. And since the whole project is available as a zipped file, it can even be placed on a local server by simply downloading and unzipping one file [20].

The actual substructure and similarity search is performed by a JavaScript engine generated by translating the original Java code developed at Actelion. This Java code is also freely available as part of the open-source OpenChemLib project [21] and as part of the open-source cheminformatics visualization tool DataWarrior [22]. As mentioned earlier, every Wikipedia molecule entry contains a 512 bit binary fingerprint, the FragFp descriptor, which is also used as the default descriptor in DataWarrior. This fragment dictionary based descriptor encodes for every structure, which of 512 predefined fragments are present in it. Whenever the query structure is updated by the user, its FragFp descriptor is calculated on-the-fly by running 512 substructure searches on the query structure. If the search mode is set to compound similarity, then the similarity of all Wikipedia structures to the query structure is determined as Tanimoto similarity between the two respective FragFp descriptors. In case of substructure search mode a two-step search is performed. In the first step all compounds are selected, whose FragFp descriptors contain at least those bits that constitute the query fingerprint. The rationale behind this is that every substructure of the query must as well be a substructure of a superstructure of the query. In a second step all selected Wikipedia compounds are checked by a graph-matching algorithm, whether they contain the query structure as a subgraph. If the search mode is equivalence then the query structure is converted into a canonical representation and compared to the canonical encoding of the Wikipedia structures.

The search is interactive. This means that each time the query is modified by adding or removing atom or bond a new search is performed and the results are immediately displayed. In order to provide more intuitive results, particularly for very small queries, simple tuning has been implemented. For substructure search the results are sorted by absolute mass difference between molecular weight and the query molecular weight while similarity search hits having the same score are sorted by the absolute difference of molecular weight with the query and checking that the exact match is always on the top of the list. These simple “tricks” assure that the query structure will always be the first hit which is the result a user would expect.

## Results and discussion

**Figure 4:**
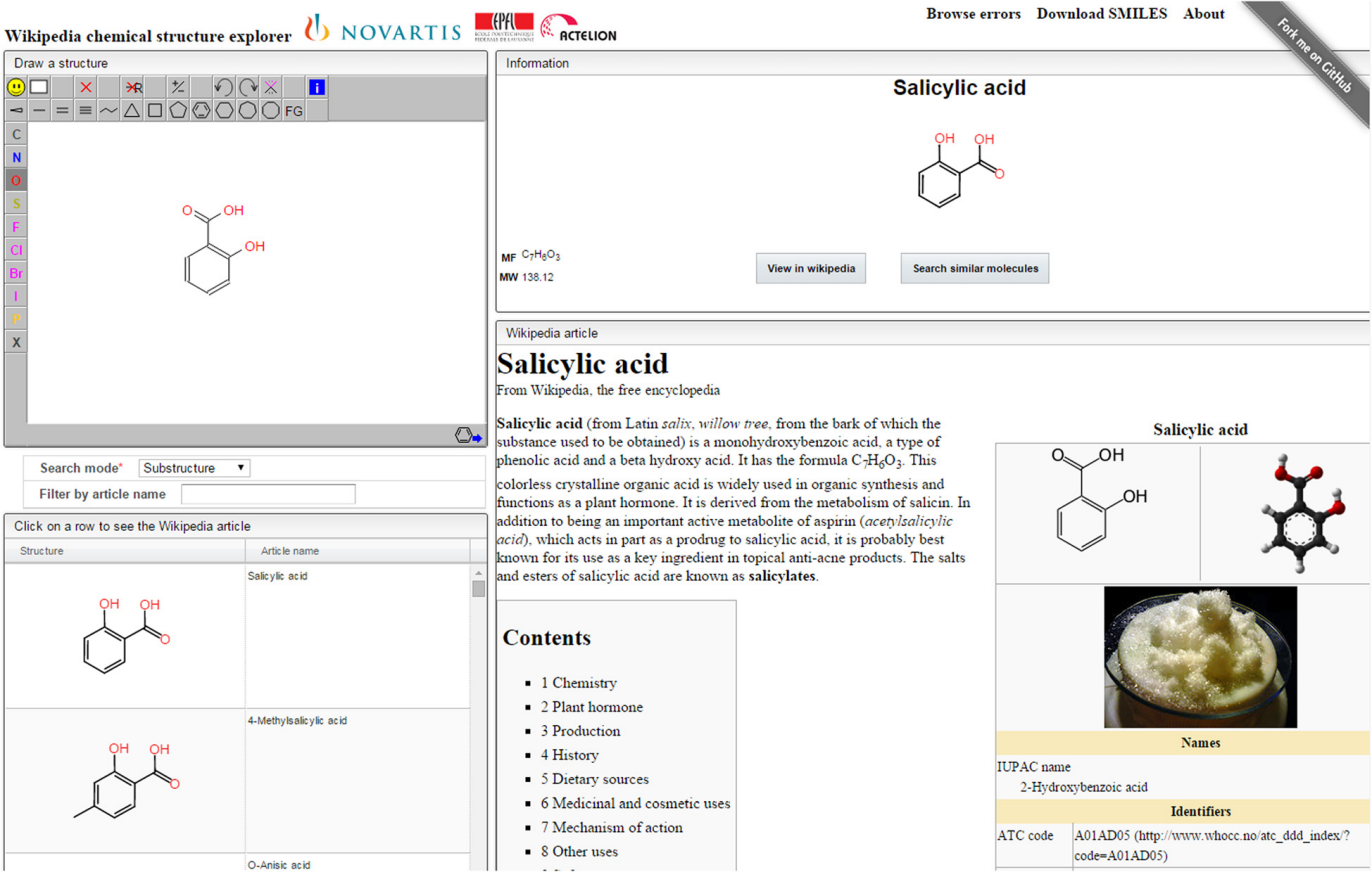
**Wikipedia Chemical Structure Explorer interface.**

The user interface (Figure 4) consists of several modules. The JSME editor [15] in the top-left module allows to draw a query structure easily. Below is a selector for the search mode (substructure, similarity or exact match) and a text field that allows to filter the results by keeping only molecules whose names contain the entered text. Matching molecules are sorted by relevance and displayed in a table with the structure and name. Chemical structure images are generated by JavaScript code that was also derived from the OpenChemLib project [23]. First the SMILES code is converted into a chemical graph, then 2-dimensional atom coordinates are generated and bonds and atom labels are drawn esthetically optimized considering details like aromaticity and implicit hydrogens. The actual molecule display, where the molecule is rendered on a HTML5 canvas using a JavaScript library, is done by using a structure drawing package from Actelion [21]. The original Java source code has been compiled via GWT into the corresponding JavaScript files.

More information about hits may be obtained by moving mouse over a result line. Clicking on the hit loads the corresponding Wikipedia page directly in the bottom-right module. The page may be opened also in a new window.

## Conclusions

We developed a web-based Wikipedia Chemical Structure Explorer allowing easy, user friendly substructure and similarity searching and navigation within the Wikipedia chemical content. The tool provides a useful resource for research as well as for chemical education. The presented analysis can hopefully also help to improve quality of chemical entries in Wikipedia by providing daily updated list of entries with problematic or missing structural information and directing in this way potential contributors to the area where they effort is mostly needed. And last but not least this search system is a nice showcase example of how the modern web technology can be applied in the field of cheminformatics. The Wikipedia Chemical Structure Explorer is available at www.cheminfo.org/wikipedia and the code is available also from GitHub https://github.com/cheminfo/wikipedia for local installation. The system is released under the open source BSD license.

## Availability and requirements

- **Project name:** Wikipedia Chemical Structure Explorer.
- **Project home page:**http://www.cheminfo.org/wikipedia/
- **Operating system:** Platform independent.
- **Programming language:** JavaScript.
- **Other requirements:** none.
- **License:** the tool itself BSD, various support libraries have their own licenses.
- **Any restrictions to use by non-academics:** none other than those specified by the licenses.
